# Supplementary material for: GSG1L-containing AMPA receptor complexes are defined by their spatiotemporal expression, native interactome and allosteric sites
Source: Nat Commun. 2023 Oct 26;14:6799. doi: 10.1038/s41467-023-42517-7 (PMC10603098; doi:10.1038/s41467-023-42517-7)
Supplement: Supplementary file 1 — Supplementary Information [file 41467_2023_42517_MOESM1_ESM.pdf]

Supplementary information for:

GSG1L-containing AMPA receptor complexes are defined by their spatiotemporal expression, native interactome and allosteric sites

Amanda M. Perozzo, Jochen Schwenk, Aichurok Kamalova, Terunaga Nakagawa, Bernd Fakler, and Derek Bowie*

*Correspondence to: derek.bowie@mcgill.ca

Containing:
Supplementary Figures 1-8
Supplementary Tables 1-5

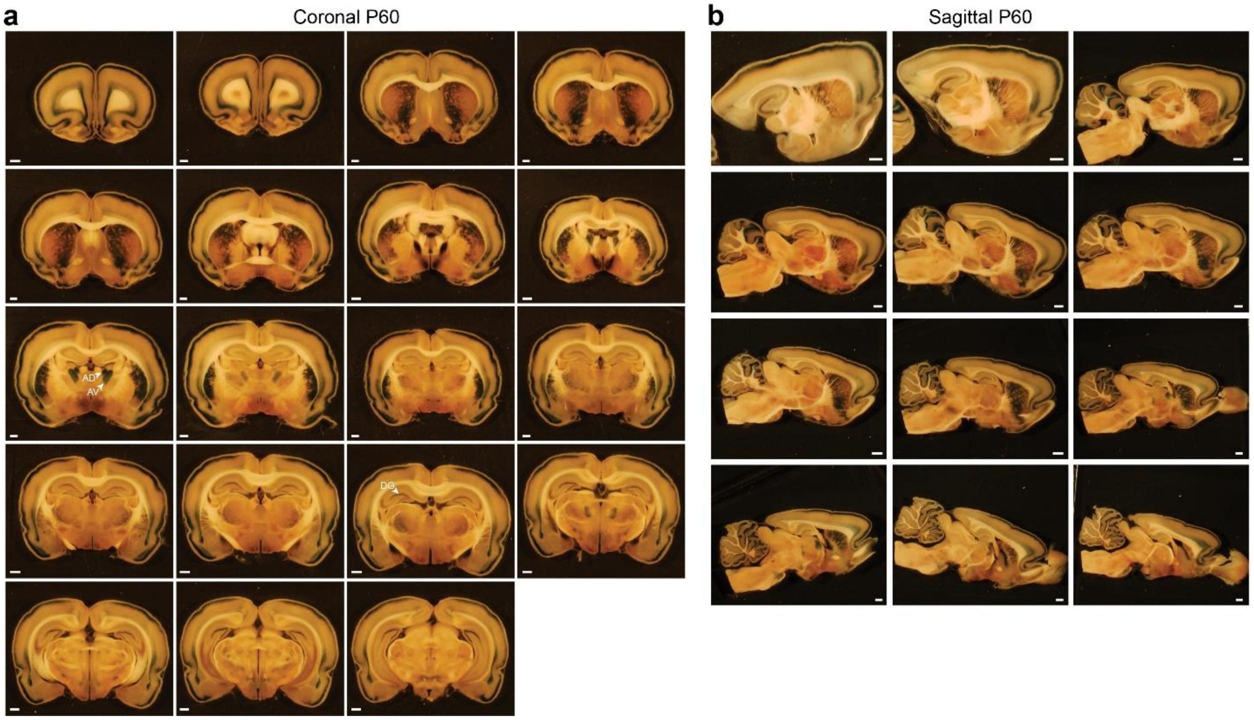

Supplementary Fig. 1: GSG1L expression in the adult rat brain.
a-b GSG1L KO rat brain (**a**) coronal and (**b**) sagittal sections at P60 (scale bar = 1000 μm, n=2). The dark blue staining is indicative of lacZ expression, which serves as a reporter for GSG1L promoter activity. The anterodorsal (AD) and anteroventral (AV) nuclei of the anterior thalamus, as well as the dentate gyrus (DG) of the hippocampus are labelled. Related to **Fig. 1**.

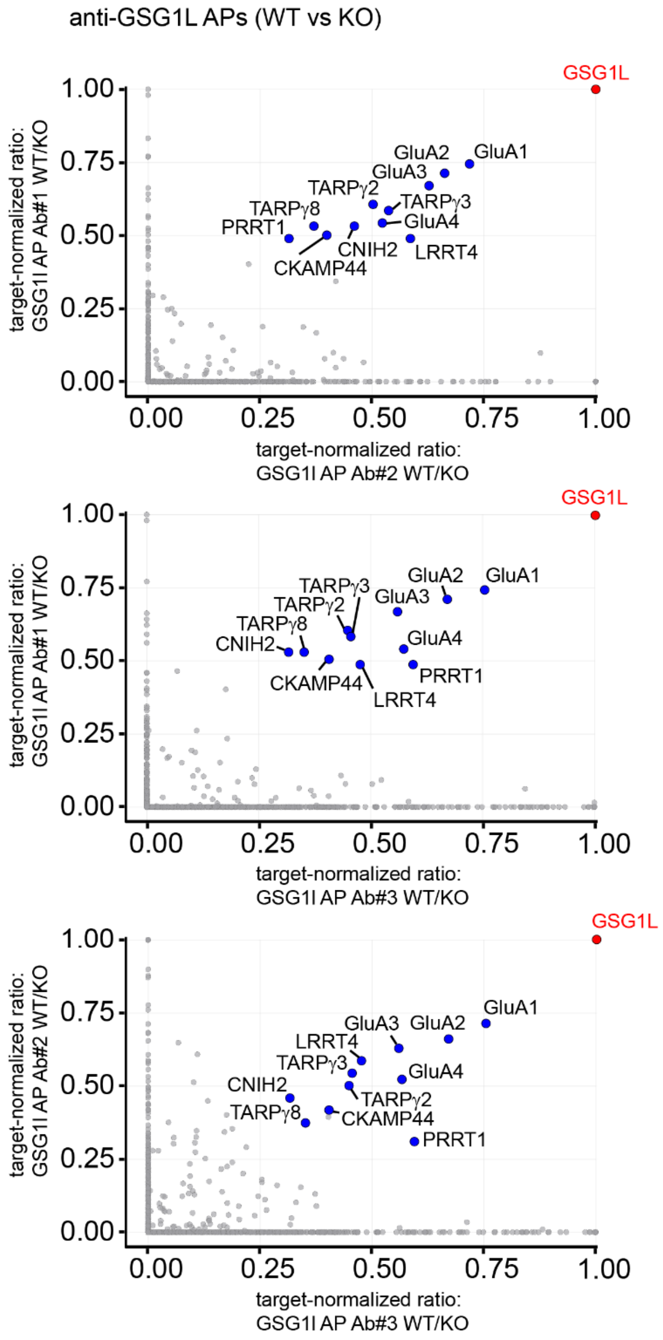

Supplementary Fig. 2: Identification of GSG1L interactors in the adult rat brain.
Abundances of all proteins identified in GSG1L APs, obtained with three distinct anti-GSG1L antibodies from solubilized WT and GSG1L KO rat brain (P59) membranes, were determined and abundance ratios calculated (tnR-values; see **Methods**). Ratio plots show specific (tnR > 0.25) and highly consistent co-purification of GluA1-4 proteins and a set of their proteome constituents. Related to **Fig. 2**.

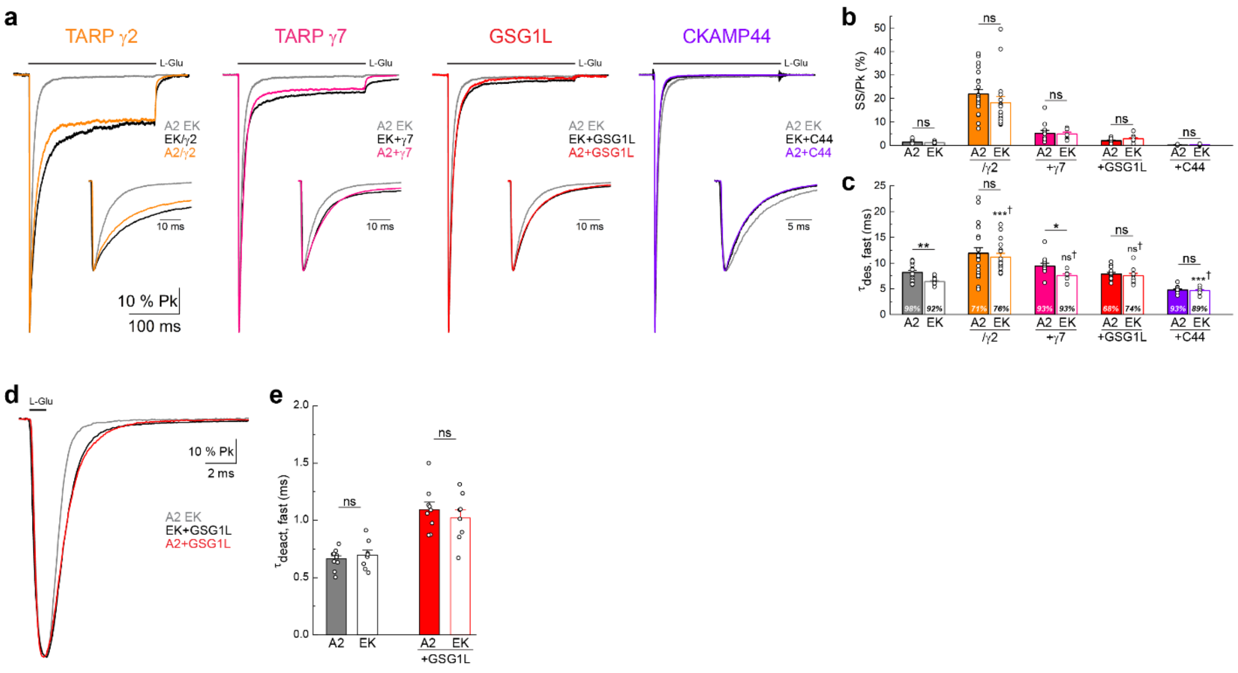

Supplementary Fig. 3: Mutation of residue E658 in GluA2 does not affect GSG1L modulation of AMPAR gating kinetics.

a Scaled current responses of GluA2 E658K (grey, patch 180522p4) expressed with γ2 (patch 180517p1), γ7 (patch 180723p4), GSG1L (patch 180723p3) and CKAMP44 (patch 180626p6) (from left to right, black) upon a 250 ms application of 10 mM L-Glu. GluA2 WT (colored trace, previously presented in **Fig. 3**) is shown for reference. Insets depict the onset of the response on a shorter time-scale. **b** Mean equilibrium current amplitude as a percentage of the peak response. **c** Time constants for the fast component of desensitization ($\tau_{des,\ fast}$). Percentage on bars indicates the individual contribution of the fast component to the overall current decay. For (**b-c**), A2 data were first shown in **Fig. 3d-e**. Data are mean ± SEM, where n=12 for GluA2 EK, n=17 for EK/γ2, n=7 for EK+γ7, n=10 for EK+GSG1L and n=8 for EK+CKAMP44. (ns = not significant, *p < 0.05, **p < 0.01, unpaired two-tailed Student's t-test or Mann-Whitney U test. ns† = not significant, ***†p < 0.001, compared to EK alone, Kruskal-Wallis ANOVA followed by Mann-Whitney U tests with Bonferroni-Holm correction). **d** Scaled current responses of GluA2 E658K (grey, patch 180510p11) expressed with GSG1L (black, patch 180723p3) in response to a 1 ms application of 10 mM L-Glu. The WT receptor (GluA2 expressed with GSG1L) is shown for reference (red, patch 180423p2). **e** Time constants for the fast component of deactivation ($\tau_{deact,\ fast}$). Data are mean ± SEM, where n=10 for GluA2, n=8 for GluA2 EK, n=9 for A2+GSG1L and n=8 for EK+GSG1L. (ns = not significant, unpaired two-tailed Student's t-test). For (**b-c, e**), source data are provided as a Source Data file. Related to **Fig. 5**; see also **Supplementary Table 1**.

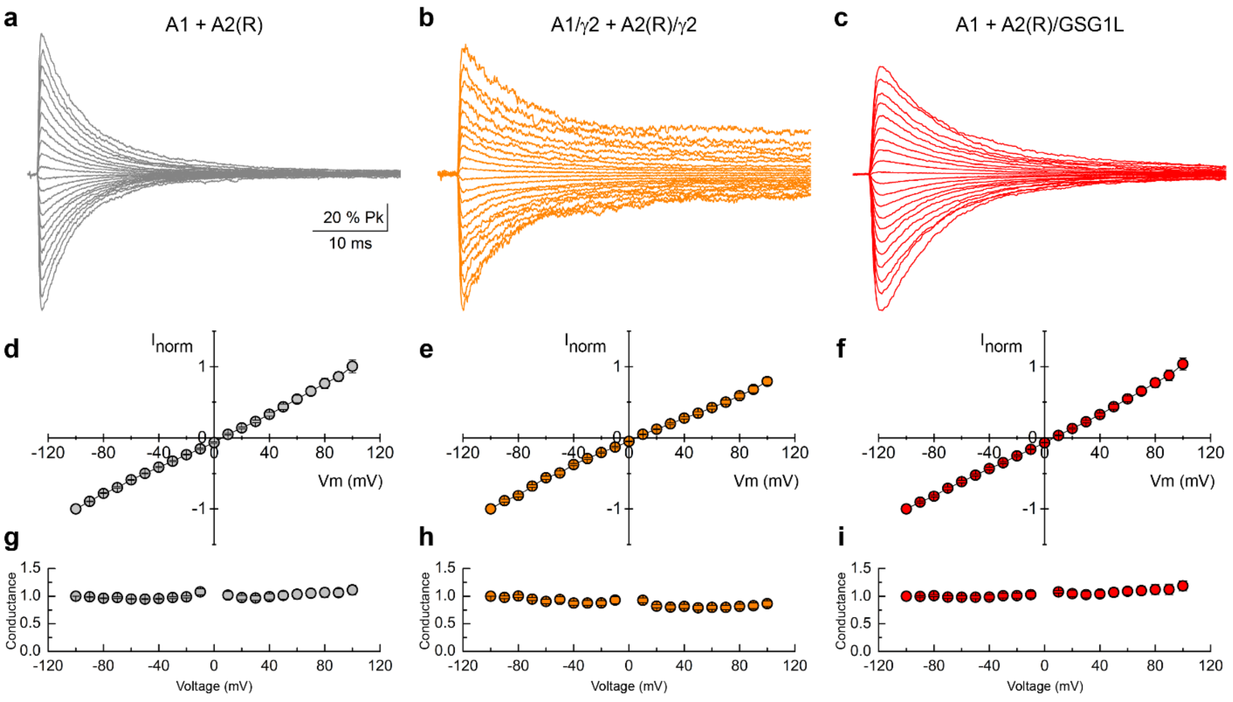

Supplementary Fig. 4: GluA2(R)-containing heteromers are insensitive to channel block by intracellular polyamines.

a-c Example traces from (**a**) GluA1 + GluA2(R) (patch 191001p8), (**b**) GluA1/γ2 + GluA2(R)/γ2 (patch 190822p11), and (**c**) GluA1 + GluA2(R)/GSG1L (patch 200811p11) exposed to 10 mM L-Glu (250 ms) at different membrane potentials (range from -100 to +100 mV, 10 mV increments) in the presence of 30 μM internal spermine (Spm). **d-f** Mean I-V plots, normalized to -100 mV in Spm, for the receptor complexes shown in panels (**a-c**). **g-i** Mean G-V plots for the receptor complexes shown in panels (**a-c**). Data are mean ± SEM. Related to **Figs. 6** and **7**.

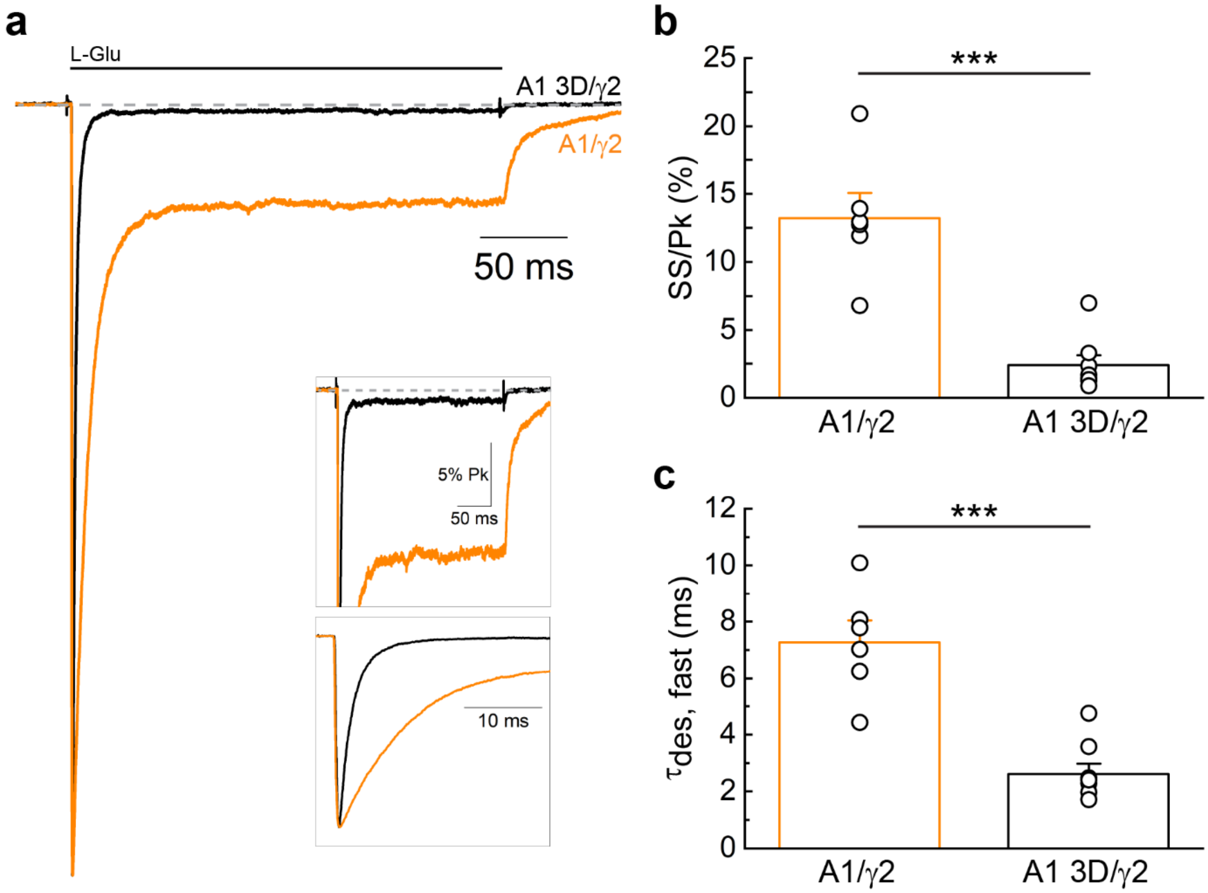

Supplementary Fig. 5: Mutation of the KGK motif in GluA1 homomers attenuates TARP γ2 modulation of desensitization.

a Scaled current responses of GluA1/γ2 (patch 190829p2) and GluA1 3D/γ2 (patch 190829p8) upon a 250 ms application of 10 mM L-Glu. Insets depict the equilibrium response (top) and the onset of the response on a shorter timescale (bottom). **b** Mean equilibrium current amplitude as a percentage of the peak response. **c** Time constants for the fast component of desensitization ($\tau_{des,\ fast}$). Data are mean ± SEM, where n=6 for GluA1/γ2 and n=8 for GluA1 3D/γ2. (***$p \leq 0.001$, (**b**) Mann-Whitney U test, (**c**) unpaired Student's t-test). Source data are provided as a Source Data file. Related to **Fig. 6**; see also **Supplementary Table 3**.

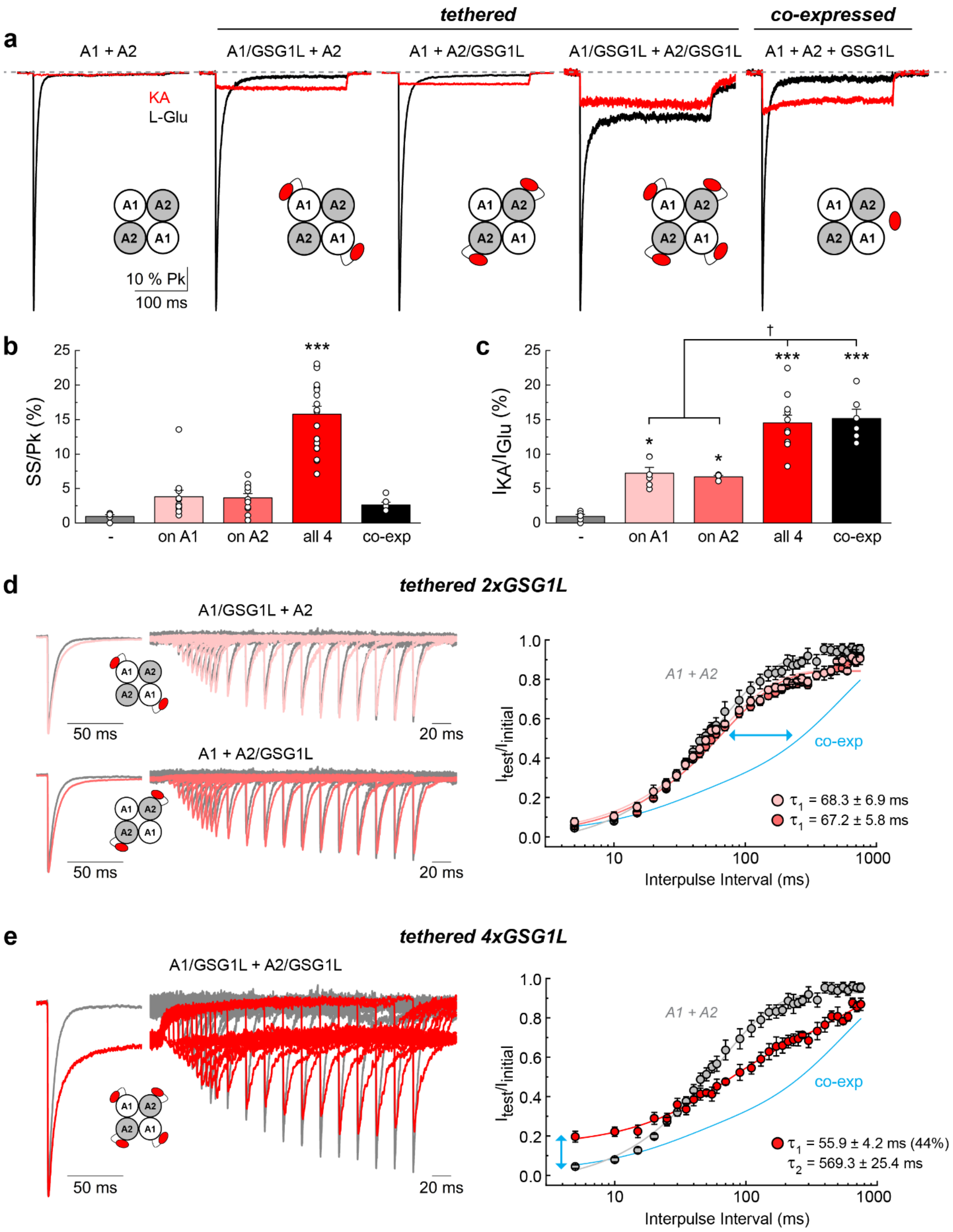

a
tethered
co-expressed
A1 + A2
A1/GSG1L + A2
A1 + A2/GSG1L
A1/GSG1L + A2/GSG1L
A1 + A2 + GSG1L
KA
L-Glu
10 % Pk
100 ms
b
SS/Pk (%)
-
on A1
on A2
all 4
co-exp
***
c
I_{KA}/I_{Glu} (%)
†
*
***
d
tethered 2xGSG1L
50 ms
20 ms
A1 + A2
co-exp
$I_{test}/I_{initial}$
Interpulse Interval (ms)
τ_1 = 68.3 ± 6.9 ms
τ_1 = 67.2 ± 5.8 ms
e
tethered 4xGSG1L
τ_1 = 55.9 ± 4.2 ms (44%)
τ_2 = 569.3 ± 25.4 ms

Supplementary Fig. 6: AMPARs tethered to or co-expressed with GSG1L subunits exhibit distinct properties.

a Scaled current responses of A1/A2 heteromers evoked by 250 ms applications of 10 mM L-Glu (black) or 1 mM KA (red). From left to right: 0xGSG1L (patch 200714p5), 2xGSG1L with GSG1L tethered to A1 (patch 200810p2) or A2 (patch 200811p11), 4xGSG1L with GSG1L tethered to all GluA subunits (patch 200717p2) and co-expressed GSG1L (patch 200716p6). Co-expression data also appears in **Fig. 7a$_2$**. **b** Mean equilibrium current amplitude as a percentage of the peak response. Data are mean ± SEM where n=5 for A1/A2, n=12 for A1/GSG1L, n=11 for A2/GSG1L, n=19 for 4xGSG1L and n=7 for co-expressed GSG1L. (***p < 0.001 4xGSG1L vs. all other receptor combinations, one-way ANOVA with Tukey's HSD test). Only significant results are indicated for clarity. **c** Mean KA current amplitude as a percentage of the peak response in L-Glu**.** Data are mean ± SEM where n=5 for A1/A2, n=6 for A1/GSG1L, n=4 for A2/GSG1L, n=11 for 4xGSG1L and n=6 for co-expressed GSG1L. (*p < 0.05, ***p < 0.001 compared to A1/A2 alone; †p ≤ 0.001 for on A1 vs. all 4, on A1 vs. co-exp, on A2 vs. all 4, and on A2 vs. co-exp, one-way ANOVA with Tukey's HSD test). Only significant results are indicated for clarity. For (**b-c**), source data are provided as a Source Data file. **d** Recovery from desensitization for A1/A2 heteromers with GSG1L tethered to either GluA1 (top, patch 200810p2, light pink) or GluA2 (bottom, patch 200810p17, dark pink). The scatter plot on the right depicts the recovery time course and time constants ($\tau_{recovery}$) for each receptor combination. The solid lines represent average fits of the data. Data are mean ± SEM where n=7 for A1/GSG1L (light pink) and n=8 for A2/GSG1L (dark pink). Control data was previously presented in **Fig. 7a$_1$**. **e** Recovery from desensitization for A1/A2 heteromers with GSG1L tethered to both GluA1 and GluA2 (patch 200619p13, red). The scatter plot on the right depicts the recovery time course and time constants ($\tau_{recovery}$). The solid lines represent average fits of the data. Data are mean ± SEM where n=6 for A1/GSG1L + A2/GSG1L (red). Control data was previously presented in **Fig. 7a$_1$**. Related to **Fig. 7**; see also **Supplementary Tables 3-5** and **Methods**.

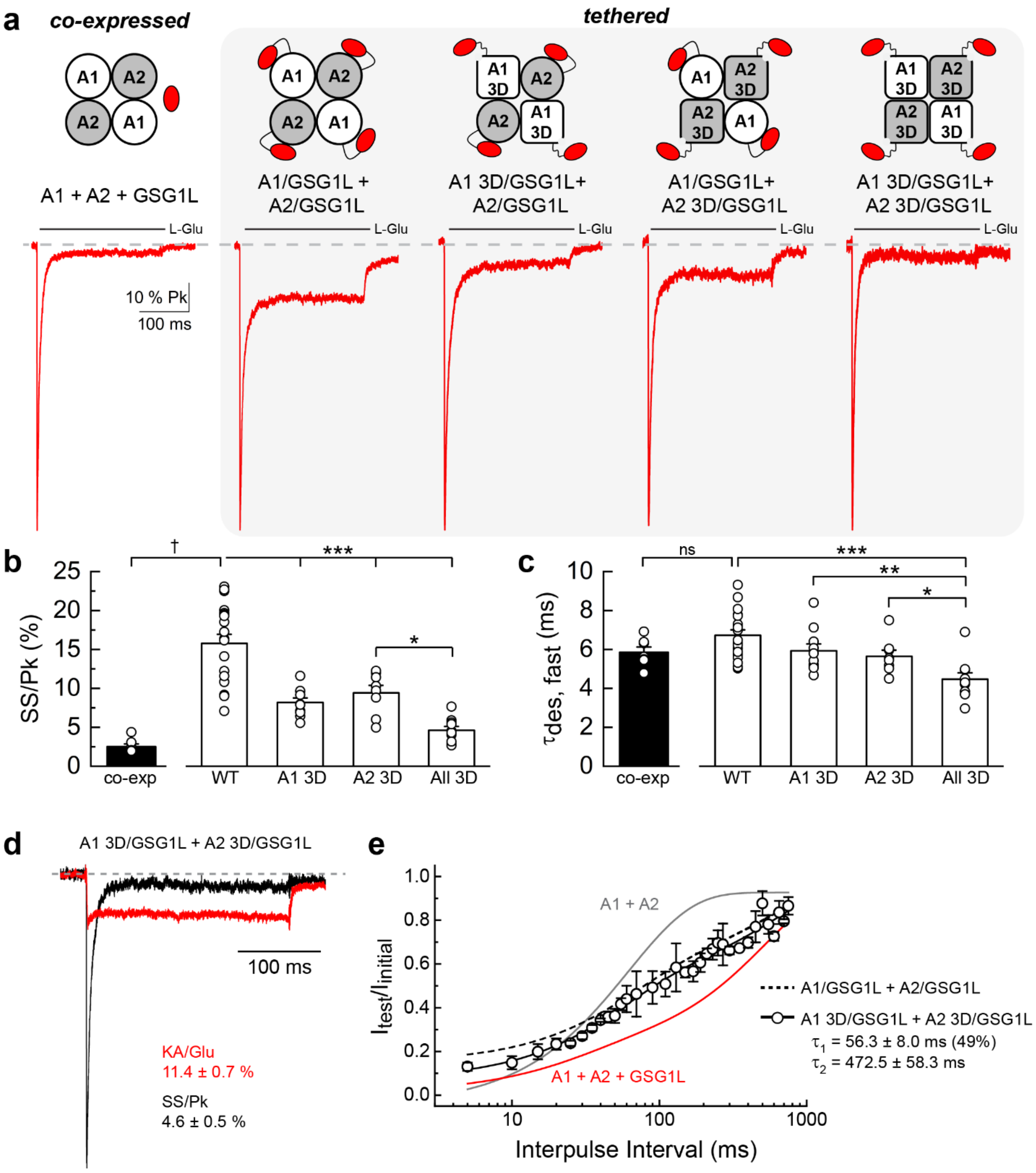
a
co-expressed
tethered
A1 + A2 + GSG1L
A1/GSG1L +
A2/GSG1L
A1 3D/GSG1L+
A2/GSG1L
A1/GSG1L+
A2 3D/GSG1L
A1 3D/GSG1L+
A2 3D/GSG1L
L-Glu
10 % Pk
100 ms
b
SS/Pk (%)
co-exp
WT
A1 3D
A2 3D
All 3D
c
τdes, fast (ms)
ns
d
A1 3D/GSG1L + A2 3D/GSG1L
100 ms
KA/Glu
11.4 ± 0.7 %
SS/Pk
4.6 ± 0.5 %
e
A1 + A2
A1/GSG1L + A2/GSG1L
A1 3D/GSG1L + A2 3D/GSG1L
τ1 = 56.3 ± 8.0 ms (49%)
τ2 = 472.5 ± 58.3 ms
A1 + A2 + GSG1L
Itest/Iinitial
Interpulse Interval (ms)

Supplementary Fig. 7: Heteromers tethered to 4xGSG1L subunits exhibit an enhanced steady-state response that is mediated through the KGK motif.

a Scaled current responses of A1/A2 heteromers co-expressed with or tethered to GSG1L subunits in response to a 250 ms application of 10 mM L-Glu. From left to right: co-expressed (co-exp, patch 200716p6), wildtype tethered heteromer (WT, patch 200717p2), 3D mutation on GluA1 (A1 3D, patch 200608p11), 3D mutation on GluA2 (A2 3D, patch 200928p8) and 3D mutation on all pore-forming subunits (All 3D, patch 200929p1). WT tethered data are also shown in **Supplementary Fig. 6**. **b** Mean equilibrium current amplitude as a percentage of the peak response. **c** Time constants for the fast component of desensitization ($\tau_{des,\ fast}$). For (**b-c**), data are mean ± SEM where n=19 for WT, n=10 for A1 3D, n=8 for A2 3D and n=10 for All 3D. (ns = not significant, $^{\dagger}p < 0.001$, unpaired two-tailed Student's t-test co-exp vs. WT; *p < 0.05, **p < 0.01, ***p < 0.001, (**b**) one-way ANOVA with Tukey's HSD test, (**c**) Kruskal-Wallis ANOVA followed by Mann-Whitney U tests with Bonferroni-Holm correction). Only significant results are indicated for clarity for tethered receptors. For (**b-c**), source data are provided as a Source Data file. **d** Same as (**a**), right-most receptor combination (i.e., A1/A2 heteromer with 3D mutation on both GluA1 and GluA2 tethered to GSG1L subunits) to show the KA-evoked response (1 mM, red). The steady-state current in L-Glu and KA as a percentage of the peak response in L-Glu is indicated (mean ± SEM, n=6). **e** Recovery from desensitization time course and time constants ($\tau_{recovery}$) for the receptors shown in (**d**). The solid and dashed lines represent average fits of the data for the indicated combinations. Data are mean ± SEM where n=4 for A1 3D/GSG1L + A2 3D/GSG1L (open circles). Related to **Fig. 7**; see also **Supplementary Tables 3-5** and **Methods**.

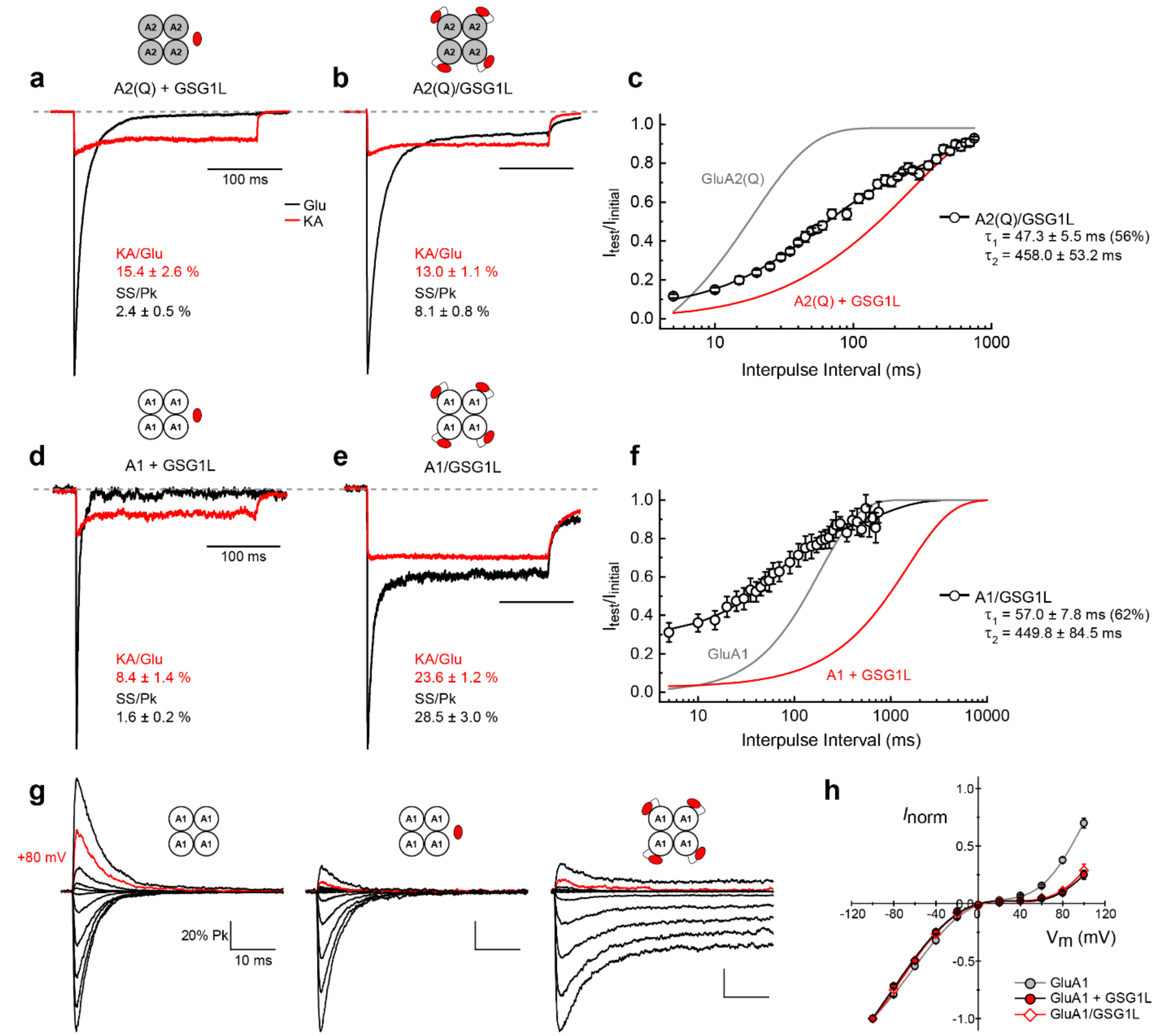

a
A2(Q) + GSG1L
b
A2(Q)/GSG1L
c
100 ms
Glu
KA
KA/Glu
15.4 ± 2.6 %
SS/Pk
2.4 ± 0.5 %
KA/Glu
13.0 ± 1.1 %
SS/Pk
8.1 ± 0.8 %
GluA2(Q)
A2(Q) + GSG1L
A2(Q)/GSG1L
τ1 = 47.3 ± 5.5 ms (56%)
τ2 = 458.0 ± 53.2 ms
Itest/Iinitial
Interpulse Interval (ms)
d
A1 + GSG1L
e
A1/GSG1L
f
100 ms
KA/Glu
8.4 ± 1.4 %
SS/Pk
1.6 ± 0.2 %
KA/Glu
23.6 ± 1.2 %
SS/Pk
28.5 ± 3.0 %
GluA1
A1 + GSG1L
A1/GSG1L
τ1 = 57.0 ± 7.8 ms (62%)
τ2 = 449.8 ± 84.5 ms
Itest/Iinitial
Interpulse Interval (ms)
g
+80 mV
20% Pk
10 ms
h
Inorm
Vm (mV)
GluA1
GluA1 + GSG1L
GluA1/GSG1L

Supplementary Fig. 8: Tethered homomeric AMPAR-GSG1L complexes exhibit some similar but also some different biophysical properties compared to homomers co-expressed with free GSG1L. **a** Scaled current response of GluA2 homomers co-expressed with GSG1L (patch 200716p17) upon application of 10 mM L-Glu (black) or 1 mM KA (red). Data represents n=6. **b** Same as (**a**) but GSG1L is tethered to GluA2 (patch 200831p16). Data represents n=6. For (**a-b**), source data are provided as a Source Data file. **c** Scatter plot depicts the recovery time course and time constants ($\tau_{recovery}$) for the receptor combination indicated. The solid lines represent average fits. Data are mean ± SEM where n=6 for GluA2/GSG1L (tethered). **d** Scaled current response of GluA1 homomers co-expressed with GSG1L (patch 210507p4) upon application of 10 mM L-Glu (black) or 1 mM KA (red). Data represents n=9. **e** Same as (**d**) but GSG1L is tethered to GluA1 (patch 200710p1). Data represents n=5. For (**d-e**), source data are provided as a Source Data file. **f** Scatter plot depicts the recovery time course and time constants ($\tau_{recovery}$) for the receptor combination indicated. The solid lines represent average fits. Data are mean ± SEM where n=6 for GluA1/GSG1L (tethered). **g** Example traces from GluA1 receptors alone (left, patch 210518p9), co-expressed with free GSG1L (middle, patch 210514p5), and tethered to GSG1L (right, patch 200710p2) exposed to 10 mM L-Glu (250 ms) at different membrane potentials (range from -100 to +100 mV, 10 mV increments) in the presence of 30 µM internal spermine (Spm). **h** Mean I-V plots, normalized to -100 mV in Spm, for the receptor complexes shown in (**g**). Whether tethered or freely expressed, GSG1L similarly enhances polyamine channel block of GluA1 receptors. Related to **Fig. 7**; see also **Supplementary Tables 2-3, 5** and **Methods.**

Supplementary Table 1: Decay kinetics of wildtype and mutant GluA2(Q) homomeric receptors in the absence and presence of auxiliary subunits.

	$I_{equilibrium}$ (%)	τ_{fast} (ms)	τ_{slow} (ms)	% fast	$\tau_{weighted}$ (ms)	n
GluA2 WT						
**Alone**						
Desensitization	1.4 ± 0.2	8.2 ± 0.4	48.0 ± 5.4	97.7 ± 1.4	**8.5 ± 0.4**	20
Deactivation		0.66 ± 0.03	5.3 ± 1.1	99.4 ± 0.4	**0.69 ± 0.03**	10
**/γ2**						
Desensitization	22.2 ± 1.9	12.0 ± 1.1	52.3 ± 10.9	70.7 ± 4.9	**18.8 ± 1.3**	23
Deactivation		0.95 ± 0.07	9.1 ± 0.8	87.5 ± 1.7	**1.9 ± 0.2**	17
**+γ7**						
Desensitization	5.2 ± 1.3	9.5 ± 0.6	64.5 ± 8.3	92.9 ± 2.1	**12.3 ± 0.9**	11
Deactivation		0.81 ± 0.05	9.9 ± 1.4	99.9 ± 0.1	**0.83 ± 0.05**	9
**+GSG1L**						
Desensitization	2.1 ± 0.3	7.9 ± 0.3	25.0 ± 1.6	67.0 ± 2.6	**13.4 ± 0.7**	15
Deactivation		1.1 ± 0.1	5.3 ± 0.5	95.5 ± 1.1	**1.3 ± 0.1**	9
**+CKAMP44**						
Desensitization	0.25 ± 0.05	4.8 ± 0.2	15.4 ± 1.3	92.7 ± 1.5	**5.6 ± 0.2**	11
Deactivation		0.58 ± 0.05	6.6 ± 1.6	99.7 ± 0.3	**0.58 ± 0.05**	7
GluA2 3D						
**Alone**						
Desensitization	1.2 ± 0.2	5.9 ± 0.2	49.4 ± 15.5	97.4 ± 0.9	**6.4 ± 0.2**	18
Deactivation		0.56 ± 0.05	5.5 ± 0.6	99.1 ± 0.3	**0.59 ± 0.07**	8
**/γ2**						
Desensitization	5.3 ± 0.7	7.9 ± 0.7	29.4 ± 3.7	77.3 ± 3.4	**11.7 ± 0.9**	25
Deactivation		0.71 ± 0.05	8.9 ± 1.1	98.4 ± 0.5	**0.82 ± 0.05**	6
**+γ7**						
Desensitization	0.92 ± 0.23	5.1 ± 0.3	33.6 ± 5.0	97.4 ± 0.5	**5.8 ± 0.4**	7
Deactivation		0.54 ± 0.04	6.7 ± 0.5	99.9 ± 0.1	**0.54 ± 0.04**	7
**+GSG1L**						
Desensitization	0.75 ± 0.24	6.4 ± 0.4	21.8 ± 2.3	91.0 ± 2.2	**7.4 ± 0.3**	11
Deactivation		0.78 ± 0.03	3.8 ± 0.7	97.7 ± 1.1	**0.83 ± 0.03**	11
**+CKAMP44**						
Desensitization	0.27 ± 0.08	4.0 ± 0.2	16.4 ± 2.4	93.1 ± 1.6	**4.7 ± 0.3**	6
Deactivation		0.50 ± 0.02	5.9 ± 0.5	99.8 ± 0.2	**0.50 ± 0.02**	6
GluA2 E658K						
**Alone**						
Desensitization	1.2 ± 0.1	6.4 ± 0.2	41.3 ± 5.4	92.0 ± 1.3	**8.8 ± 0.4**	12
Deactivation		0.70 ± 0.04	7.0 ± 0.5	99.3 ± 0.3	**0.74 ± 0.04**	8
**/γ2**						
Desensitization	18.2 ± 2.7	11.2 ± 0.7	56.2 ± 4.3	75.6 ± 2.0	**22.2 ± 2.0**	17
Deactivation		1.09 ± 0.05	10.3 ± 0.5	91.4 ± 1.9	**1.9 ± 0.2**	12
**+γ7**						
Desensitization	4.9 ± 0.9	7.5 ± 0.4	69.8 ± 6.9	93.1 ± 0.7	**11.8 ± 0.7**	7
Deactivation		0.81 ± 0.01	9.8 ± 0.5	99.2 ± 0.2	**0.89 ± 0.02**	6
**+GSG1L**						
Desensitization	2.8 ± 0.5	7.5 ± 0.5	24.7 ± 1.8	73.8 ± 3.1	**11.8 ± 0.8**	10
Deactivation		1.0 ± 0.1	8.7 ± 0.5	93.9 ± 1.5	**1.5 ± 0.1**	8
**+CKAMP44**						
Desensitization	0.35 ± 0.10	4.6 ± 0.2	19.8 ± 1.5	88.6 ± 1.3	**6.2 ± 0.3**	8
Deactivation		0.61 ± 0.05	8.5 ± 1.0	98.6 ±0.3	**0.7 ± 0.1**	5

Receptors were activated by long (250 ms) or short (1 ms) applications of 10 mM L-glutamate (L-Glu) to measure desensitization or deactivation kinetics, respectively. Weighted time constants ($\tau_{weighted}$) were calculated based on the relative area fit by the fast and slow components. Time constants are listed in milliseconds (ms). $I_{equilibrium}$ refers to the steady-state current as a percentage of the peak response for long pulses. A "/" denotes tethered constructs, while "+" denotes co-expression. The number of patches for each condition (n) is indicated, and all values represent mean ± SEM.

Supplementary Table 2: Recovery from desensitization kinetics of wildtype and mutant GluA1 and GluA2(Q) homomeric receptors in the absence and presence of auxiliary subunits.

	τ_1 (ms)	%A_1	τ_2 (ms)	Weighted $\tau_{recovery}$ (ms)	n
GluA2 WT					
**Alone**	22.3 ± 2.0	100	-	**22.3 ± 2.0**	10
**/γ2**	16.7 ± 0.9	100	-	**16.7 ± 0.9**	6
**+GSG1L**	37.5 ± 5.4	16.0	322.5 ± 34.0	**270.2 ± 23.3**	9
**/GSG1L**	47.3 ± 5.5	55.7	458.0 ± 53.2	**224.6 ± 13.7**	6
**+CKAMP44**	138.4 ± 19.4	100	-	**138.4 ± 19.4**	8
GluA2 3D					
**Alone**	20.4 ± 1.7	100	-	**20.4 ± 1.7**	6
**/γ2**	32.5 ± 1.7	100	-	**32.5 ± 1.7**	17
**+GSG1L**	50.5 ± 12.0	15.9	300.2 ± 23.7	**252.5 ± 16.0**	7
**+CKAMP44**	148.9 ± 23.0	100	-	**148.9 ± 23.0**	5
GluA2 E658K					
**Alone**	47.0 ± 3.3	100	-	**47.0 ± 3.3**	6
**/γ2**	32.0 ± 2.6	100	-	**32.0 ± 2.6**	7
**+GSG1L**	48.0 ± 4.6	100	-	**48.0 ± 4.6**	8
**+CKAMP44**	180.1 ± 18.0	100	-	**180.1 ± 18.0**	8
GluA1 WT					
**Alone**	175.9 ± 13.9	100	-	**175.9 ± 13.9**	7
**+GSG1L**	1493.9 ± 77.9	100	-	**1493.9 ± 77.9**	9
**/GSG1L**	57.0 ± 7.8	62.3	449.8 ± 84.5	**209.1 ± 58.0**	6

Receptors were first activated by a 250 ms application of 10 mM L-Glu to induce receptor desensitization. At variable intervals following the initial pulse, a second application of 10 mM L-Glu was delivered to measure recovery from desensitization. The recovery time course was fit by either a mono- or bi-exponential function, as indicated. Weighted time constants were calculated based on the relative area fit by the individual components (e.g., %A_1). Time constants are listed in milliseconds (ms). A "/" denotes tethered constructs, while "+" denotes co-expression. The number of patches for each condition (n) is indicated, and all values represent mean ± SEM.

Supplementary Table 3: Desensitization kinetics of wildtype and mutant GluA1 homomers and GluA1 + GluA2(R) heteromers in the absence and presence of auxiliary subunits.

	$I_{equilibrium}$ (%)	τ_{fast} (ms)	τ_{slow} (ms)	% fast	$\tau_{weighted}$ (ms)	n
No Auxiliary Subunits						
**A1**	0.37 ± 0.07	2.9 ± 0.1	12.5 ± 0.8	98.4 ± 0.4	**3.1 ± 0.1**	7
**A1 3D**	0.37 ± 0.08	1.9 ± 0.4	16.1 ± 3.2	99.6 ± 0.1	**2.0 ± 0.1**	7
**A1 + A2**	0.94 ± 0.25	4.6 ± 0.4	15.4 ± 1.7	91.7 ± 3.5	**5.5 ± 0.5**	5
**A1 3D + A2 3D**	0.93 ± 0.26	3.6 ± 0.2	14.9 ± 2.2	92.6 ± 2.4	**4.3 ± 0.3**	7
**A1 EK + A2 EK**	0.94 ± 0.14	4.6 ± 0.3	16.4 ± 2.0	87.1 ± 1.9	**6.1 ± 0.4**	7
TARP γ2						
**A1/γ2**	13.2 ± 1.9	7.3 ± 0.8	31.1 ± 10.3	92.5 ± 2.5	**8.6 ± 0.7**	6
**A1 3D/γ2**	2.4 ± 0.7	2.6 ± 0.4	16.4 ± 2.8	95.4 ± 1.2	**3.1 ± 0.4**	8
**A1 + A2 + γ2**	18.4 ± 2.7	6.8 ± 0.2	26.8 ± 4.7	77.7 ± 5.0	**11.0 ± 1.0**	7
**A1/γ2 + A2/γ2**	24.0 ± 2.1	7.7 ± 0.7	30.4 ± 4.3	75.9 ± 3.1	**13.4 ± 1.7**	6
**A1 3D/γ2 + A2/γ2**	25.4 ± 2.0	7.1 ± 0.4	32.7 ± 1.7	70.6 ± 2.4	**14.6 ± 0.9**	10
**A1/γ2 + A2 3D/γ2**	13.5 ± 1.1	4.9 ± 0.3	20.5 ± 1.3	67.1 ± 0.3	**9.9 ± 0.4**	9
**A1 3D/γ2 + A2 3D/γ2**	5.6 ± 0.6	4.1 ± 0.2	18.1 ± 1.6	73.8 ± 3.0	**7.7 ± 0.6**	8
GSG1L						
**A1 + GSG1L**	1.6 ± 0.2	3.7 ± 0.2	16.8 ± 2.3	92.7 ± 1.5	**4.6 ± 0.2**	11
**A1/GSG1L**	28.5 ± 3.0	6.1 ± 0.6	31.7 ± 4.7	89.5 ± 1.8	**8.4 ± 0.6**	9
**A1 + A2 + GSG1L**	2.5 ± 0.4	5.9 ± 0.3	23.8 ± 1.8	75.3 ± 5.0	**10.0 ± 0.8**	7
**A1/GSG1L + A2**	3.8 ± 1.0	6.4 ± 0.3	30.3 ± 4.6	85.5 ± 2.2	**9.1 ± 0.6**	12
**A1 + A2/GSG1L**	3.6 ± 0.6	6.1 ± 0.3	25.1 ± 2.8	87.1 ± 2.5	**8.2 ± 0.3**	11
**A1/GSG1L + A2/GSG1L**	15.8 ± 1.2	6.7 ± 0.3	35.6 ± 3.7	83.7 ± 1.5	**11.0 ± 0.5**	19
**A1 3D/GSG1L + A2/GSG1L**	8.2 ± 0.6	5.9 ± 0.4	25.4 ± 2.1	82.9 ± 4.1	**9.0 ± 0.7**	10
**A1/GSG1L + A2 3D/GSG1L**	9.4 ± 1.0	5.6 ± 0.3	28.5 ± 3.1	89.3 ± 2.3	**7.9 ± 0.4**	8
**A1 3D/GSG1L + A2 3D/GSG1L**	4.6 ± 0.5	4.5 ± 0.3	20.4 ± 3.7	87.9 ± 3.5	**5.9 ± 0.3**	10
**A1 EK + A2 + GSG1L**	2.0 ± 0.4	6.1 ± 0.5	25.7 ± 3.3	75.3 ± 2.6	**10.9 ± 1.4**	8
**A1 + A2 EK + GSG1L**	3.0 ± 0.7	7.1 ± 0.7	28.9 ±4.8	79.2 ± 2.7	**11.6 ± 1.2**	6
**A1 EK + A2 EK + GSG1L**	3.1 ± 0.4	6.4 ± 0.3	30.9 ± 2.0	77.0 ± 2.6	**12.3 ± 1.0**	7

Receptors were activated by 250 ms applications of 10 mM L-Glu to measure desensitization kinetics. $I_{equilibrium}$ refers to the steady-state current as a percentage of the peak response. Weighted time constants ($\tau_{weighted}$) were calculated based on the relative area fit by the fast and slow components. Time constants are listed in milliseconds (ms). Heteromers were identified by the inclusion of 30 μM spermine in the internal solution and analysis of linear I-V plots (see **Methods** and **Supplementary Fig. 4**). A "/" denotes tethered constructs, while "+" denotes co-expression. The number of patches for each condition (n) is indicated, and all values represent mean ± SEM.

Supplementary Table 4: Recovery from desensitization kinetics of wildtype and mutant GluA1 + GluA2(R) heteromeric receptors in the absence and presence of auxiliary subunits.

	**τ_1 (ms)**	**%A_1**	**τ_2 (ms)**	**Weighted $\tau_{recovery}$**	**n**
GluA1 + GluA2					
**Alone, A1 + A2 (WT)**	63.9 ± 7.3	100	-	**63.9 ± 7.3**	6
**Alone, A1 EK + A2 EK**	87.5 ± 6.2	100	-	**87.5 ± 6.2**	9
**A1 + A2 + GSG1L**	23.7 ± 3.8	20.1	547.6 ± 62.7	**471.4 ± 80.3**	6
**A1/GSG1L + A2**	68.3 ± 6.9	100	-	**68.3 ± 6.9**	7
**A1 + A2/GSG1L**	67.2 ± 5.8	100	-	**67.2 ± 5.8**	8
**A1/GSG1L + A2/GSG1L**	55.9 ± 4.2	44.4	569.3 ± 25.4	**343.0 ± 30.0**	6
**A1 3D/GSG1L + A2 3D/GSG1L**	56.3 ± 8.0	48.6	472.5 ± 58.3	**279.4 ± 58.8**	4
**A1 EK + A2 + GSG1L**	338.3 ± 36.7	100	-	**338.3 ± 36.7**	7
**A1 + A2 EK + GSG1L**	80.6 ± 10.7	54.7	446.0 ± 20.5	**267.5 ± 27.7**	7
**A1 EK + A2 EK + GSG1L**	129.8 ± 8.0	100	-	**129.8 ± 8.0**	9

Receptors were first activated by a 250 ms application of 10 mM L-Glu to induce receptor desensitization. At variable intervals following the initial pulse, a second application of 10 mM L-Glu was delivered to measure recovery from desensitization. The recovery time course was fit by either a mono- or bi-exponential function, as indicated. Weighted time constants were calculated based on the relative area fit by the individual components (e.g., %A_1). Time constants are listed in milliseconds (ms). A "/" denotes tethered constructs, while "+" denotes co-expression. The number of patches for each condition (n) is indicated, and all values represent mean ± SEM.

Supplementary Table 5: Kainate-evoked responses for homomeric and heteromeric AMPARs.

	KA/Glu Ratio (%)	n
GluA1		
**Alone**	0.20 ± 0.06	5
**+GSG1L**	8.4 ± 1.4	9
**/GSG1L**	23.6 ± 1.2	5
GluA2		
**Alone**	0.88 ± 0.27	6
**+GSG1L**	15.4 ± 2.6	6
**/GSG1L**	13.0 ± 1.1	6
GluA1 + GluA2 (WT)		
**Alone, A1 + A2**	0.94 ± 0.30	5
**A1 + A2 + GSG1L**	15.2 ± 1.3	6
**A1/GSG1L + A2**	7.2 ± 0.8	6
**A1 + A2/GSG1L**	6.7 ± 0.2	4
**A1/GSG1L + A2/GSG1L**	14.5 ± 1.1	11
GluA1 + GluA2 (Mutants)		
**Alone, A1 EK + A2 EK**	0.31 ± 0.07	7
**A1 EK + A2 + GSG1L**	13.2 ± 2.2	8
**A1 + A2 EK + GSG1L**	10.9 ± 0.6	6
**A1 EK + A2 EK + GSG1L**	10.9 ± 1.1	7
**A1 3D/GSG1L + A2 3D/GSG1L**	11.4 ± 0.7	6

Receptors were exposed to 250 ms applications of 10 mM L-Glu and 1 mM kainate (KA). The KA response is presented as a percentage of the peak response in L-Glu. A "/" denotes tethered constructs, while "+" denotes co-expression. The number of patches for each condition (n) is indicated, and all values represent mean ± SEM.
