# Supplementary material for: GSG1L-containing AMPA receptor complexes are defined by their spatiotemporal expression, native interactome and allosteric sites
Source: Nat Commun. 2023 Oct 26;14:6799. doi: 10.1038/s41467-023-42517-7 (PMC10603098; doi:10.1038/s41467-023-42517-7)
Supplement: Supplementary file 3 — Reporting Summary [file 41467_2023_42517_MOESM3_ESM.pdf]

Corresponding author(s): Dr. Derek Bowie
Last updated by author(s): Oct 2, 2023

Reporting Summary

Nature Portfolio wishes to improve the reproducibility of the work that we publish. This form provides structure for consistency and transparency in reporting. For further information on Nature Portfolio policies, see our Editorial Policies and the Editorial Policy Checklist.

Statistics

For all statistical analyses, confirm that the following items are present in the figure legend, table legend, main text, or Methods section.

n/a	Confirmed	
☐	☒	The exact sample size (*n*) for each experimental group/condition, given as a discrete number and unit of measurement
☐	☒	A statement on whether measurements were taken from distinct samples or whether the same sample was measured repeatedly
☐	☒	The statistical test(s) used AND whether they are one- or two-sided<br>*Only common tests should be described solely by name; describe more complex techniques in the Methods section.*
☒	☐	A description of all covariates tested
☐	☒	A description of any assumptions or corrections, such as tests of normality and adjustment for multiple comparisons
☐	☒	A full description of the statistical parameters including central tendency (e.g. means) or other basic estimates (e.g. regression coefficient) AND variation (e.g. standard deviation) or associated estimates of uncertainty (e.g. confidence intervals)
☐	☒	For null hypothesis testing, the test statistic (e.g. *F*, *t*, *r*) with confidence intervals, effect sizes, degrees of freedom and *P* value noted<br>*Give P values as exact values whenever suitable.*
☒	☐	For Bayesian analysis, information on the choice of priors and Markov chain Monte Carlo settings
☒	☐	For hierarchical and complex designs, identification of the appropriate level for tests and full reporting of outcomes
☒	☐	Estimates of effect sizes (e.g. Cohen's *d*, Pearson's *r*), indicating how they were calculated

Our web collection on statistics for biologists contains articles on many of the points above.

Software and code

Policy information about availability of computer code

Data collection	pClamp9 software (Molecular Devices, LLC)
Data analysis	ProteoWizard (http://proteowizard.sourceforge.io); Mascot 2.6.2 (Matrix Science, UK); MaxQuant v1.6.3 (http://www.maxquant.org); BELKI software suite (https://github.com/phys2/belki); Clampfit 10.5 (Molecular Devices, LLC); Origin 2020 (OriginLab)

For manuscripts utilizing custom algorithms or software that are central to the research but not yet described in published literature, software must be made available to editors and reviewers. We strongly encourage code deposition in a community repository (e.g. GitHub). See the Nature Portfolio guidelines for submitting code & software for further information.

Data

Policy information about availability of data

All manuscripts must include a data availability statement. This statement should provide the following information, where applicable:

- Accession codes, unique identifiers, or web links for publicly available datasets
- A description of any restrictions on data availability
- For clinical datasets or third party data, please ensure that the statement adheres to our policy

Data supporting the findings of this study are available within the paper and its supplementary information files. The mass spectrometry proteomics data have been deposited to the ProteomeXchange Consortium via the PRIDE partner repository (Perez-Riverol Y et al., 2022 Nucleic Acids Res.) with the dataset identifier PXD044621 and DOI 10.6019/PXD044621. Referred protein structures have the following PDB accession codes: 5WEO [https://doi.org/10.2210/pdb5weo/pdb],

5VHY [https://doi.org/10.2210/pdb5VHY/pdb], 1FTJ [https://doi.org/10.2210/pdb1FTJ/pdb], 5KBU [https://doi.org/10.2210/pdb5KBU/pdb], 7RYZ [https://doi.org/10.2210/pdb7RYZ/pdb]. Source data for Figs. 2, 3, 4, 5, 6, and 7 and Supplementary Figs. 2, 3, 5, 6, 7, and 8 are provided with the paper as indicated.

Research involving human participants, their data, or biological material

Policy information about studies with human participants or human data. See also policy information about sex, gender (identity/presentation), and sexual orientation and race, ethnicity and racism.

Reporting on sex and gender	N/A
Reporting on race, ethnicity, or other socially relevant groupings	N/A
Population characteristics	N/A
Recruitment	N/A
Ethics oversight	N/A

Note that full information on the approval of the study protocol must also be provided in the manuscript.

Field-specific reporting

Please select the one below that is the best fit for your research. If you are not sure, read the appropriate sections before making your selection.

☒ Life sciences ☐ Behavioural & social sciences ☐ Ecological, evolutionary & environmental sciences

For a reference copy of the document with all sections, see nature.com/documents/nr-reporting-summary-flat.pdf

Life sciences study design

All studies must disclose on these points even when the disclosure is negative.

Sample size	No statistical method was used to predetermine sample sizes. For histology experiments, two replicates were performed at each time point (Kamalova et al., 2020 Cell Rep.). For AP-MS experiments, brains from 3 WT and 3 GSG1L KO rats were used, similar to previous publications (Schwenk et al., 2010 Nature; Schwenk et al., 2014 Neuron). For recombinant electrophysiology experiments, data sets are in line with the standards established by the field i.e., a minimum of 4 patch recordings per transfection condition. The authors have extensive experience with data of this type and knowledge of the typical data variability (e.g., Dawe et al., 2016; 2019 Neuron; Perozzo et al., 2023 J Neurosci).
Data exclusions	No data were excluded in histology experiments. To determine GSG1L protein interactors (Fig. 2 and Supp. Fig. 2), a minimum tnR of 0.25 was used as the indicator for specific interaction (as done in other studies by the authors, e.g. Boudkkazi et al., 2023 Neuron). In electrophysiology experiments, pre-established quality control criteria common to the field (i.e., leak current, solution exchange rate) were used when analyzing patch recordings. Heteromeric receptor assemblies were evaluated and included based on linear I-V relationships (Supp. Fig. 4), as performed in Dawe et al., 2019 Neuron.
Replication	Data replicates of each experiment are detailed in the Methods and Results/figure legends. For histology, two replicates were done for each time point and the results were highly reproducible. For quantification of proteins (Fig. 2 and Supp. Fig. 2), 6 distinct anti-GSG1L APs (3 from WT and 3 controls (GSG1L KO)) provided 3 ratios. MS-derived protein abundances were successfully replicated from three independent experiments. For recombinant electrophysiology experiments, each transfection condition was performed at least two independent times and the data pooled; all results were successfully replicated.
Randomization	For histology (Fig. 1 and Supp. Fig. 1) and proteomics (Fig. 2 and Supp. Fig. 2), animals were allocated into experimental groups based on genotype (WT versus KO). Randomization is not relevant to electrophysiology experiments, as there are no experimental groups (data are organized based on known receptor composition).
Blinding	No blinding was used in this study as it was not technically or practically feasible to do so.

Reporting for specific materials, systems and methods

We require information from authors about some types of materials, experimental systems and methods used in many studies. Here, indicate whether each material, system or method listed is relevant to your study. If you are not sure if a list item applies to your research, read the appropriate section before selecting a response.

Materials & experimental systems

n/a	Involved in the study
☐	☒ Antibodies
☐	☒ Eukaryotic cell lines
☒	☐ Palaeontology and archaeology
☐	☒ Animals and other organisms
☒	☐ Clinical data
☒	☐ Dual use research of concern
☒	☐ Plants

Methods

n/a	Involved in the study
☒	☐ ChIP-seq
☒	☐ Flow cytometry
☒	☐ MRI-based neuroimaging

Antibodies

Antibodies used

anti-GSG1L antibodies: Ab#1, polyclonal, raised in rabbit against C- term of rat GSG1L (aa304-322); Ab#2, polyclonal, raised in rabbit against rat GSG1L (aa257- 278); Ab#3, polyclonal, raised in rabbit against rat GSG1L (aa287-308); Ab#4 (Proteintech, #17328-1-AP).

anti-GluA antibodies: anti-GluA1, #AB1504 Millipore; anti-GluA2, #75-002 NeuroMab; anti-GluA2/3, #07-598 Millipore; anti-GluA3, #182203 Synaptic Systems; anti-GluA4, #AB1508 Millipore.

Validation

All GSG1L antibodies used for AP-MS experiments were validated for target-specificity and efficiency by quantitative mass spectrometry (detailed in Results Fig. 2, Supp. Fig. 2 and Methods). All GluA antibodies were previously used and validated in Schwenk et al., 2012; 2014 Neuron.

Eukaryotic cell lines

Policy information about cell lines and Sex and Gender in Research

Cell line source(s)

HEK293T/17 cells (ATCC), CRL-11268

Authentication

No further authentication was performed for commercially available cell lines.

Mycoplasma contamination

Not tested specifically for this study.

Commonly misidentified lines
(See ICLAC register)

HEK cells are listed in the register; however, our cell line comes from a reliable source and is the only cell line used in this study/by our lab, which minimizes the risk of any cross-contamination.

Animals and other research organisms

Policy information about studies involving animals; ARRIVE guidelines recommended for reporting animal research, and Sex and Gender in Research

Laboratory animals

Adult (P14 to P240) WT and GSG1L KO rats; see Kamalova et al., 2020 Cell Rep. for animals related to histology experiments. Adult (P59) WT and GSG1L KO rats; see Schwenk et al., 2012 Neuron for animals related to native proteomics.

Wild animals

The study did not involve wild animals.

Reporting on sex

Sex was not considered (both sexes used).

Field-collected samples

The study did not involve samples collected from the field.

Ethics oversight

Histology: All animal procedures were approved by the Vanderbilt University Animal Care and Use Committee and were in agreement with the NIH and Vanderbilt University guidelines for the care and use of laboratory animals.

Proteomics: All animal procedures were performed according to the animal proposal X14/14H issued by the Regierungspräsidium Freiburg, Freiburg, Germany.

Note that full information on the approval of the study protocol must also be provided in the manuscript.
